# Genetic diversity of *Plasmodium falciparum* isolates from patients with uncomplicated and severe malaria based on *msp*-1 and *msp*-2 genes in Gublak, North West Ethiopia

**DOI:** 10.1186/s12936-019-3039-9

**Published:** 2019-12-10

**Authors:** Hussein Mohammed, Kedir Hassen, Ashenafi Assefa, Kalkidan Mekete, Gemechu Tadesse, Girum Taye, Robert J. Commons

**Affiliations:** 1 Ethiopian Public Health Institute, Addis Ababa, Ethiopia; 2 Menzies School of Health Research, Charles Darwin University, Darwin, Australia; 3 Internal Medical Services, Ballarat Health Services, Ballarat, Australia

**Keywords:** *Plasmodium falciparum*, Ethiopia, Genetic diversity, Merozoite surface protein, Disease severity

## Abstract

**Background:**

Malaria infection can present with a wide variety of symptoms, ranging from mild to severe. *Plasmodium falciparum* isolates in uncomplicated and severe malaria infections may have different parasite genetic profiles. This study was conducted to assess differences in genetic diversity and allelic frequencies in *P. falciparum* isolates according to malaria severity and age of patients in the Gublack area, northwest Ethiopia.

**Methods:**

Cross-sectional health facility-based study conducted in Gublak, Ethiopia between July, 2017 and October, 2017. Symptomatic *P. falciparum* malaria patients with microscopically-confirmed infection were enrolled. Parasite DNA was extracted from filter paper blood spots and the polymorphic regions of the *msp*-*1* and *msp*-*2* genes were genotyped using allele-specific nested-PCR with fragment analysis by gel electrophoresis.

**Results:**

A total of 118 patients were enrolled including 95 (80.5%) with uncomplicated infection and 23 (19.5%) with severe disease. In *msp*-1, the K1 allelic family was similarly prevalent in uncomplicated 42 (44.2%) and severe disease 12 (52.2%). In *msp*-2, FC27 was detected in 55 (57.9%) of uncomplicated infections and IC/3D7 in 14 (60.9%) of severe infections. 76 (64.4%) of the 118 isolates contained multiple genotypes; 56 (58.9%) in uncomplicated infections and 19 (82.6%) in severe infections. The overall of multiplicity of infection was 2.2 (95% CI 1.98–2.42) with 1.4 (95% CI 1.23–1.55) and 1.7 (95% CI 1.49–1.86) for *msp*-*1* and *msp*-*2*, respectively. Multiplicity of infection was significantly higher in severe than uncomplicated infections (3.0 (95% CI 2.61–3.47) versus 2.0 (95% CI 1.83–2.23), respectively, *p *= 0.001). There was no difference in multiplicity of infection across age groups (p = 0.104).

**Conclusion:**

Patients with severe malaria were more likely to have multiclonal infections. Further studies are needed to describe the association between *P. falciparum* genotypes and malaria severity in different malaria transmission areas.

## Background

Severe malaria is a major public health problem. Globally about 3.3 billion people live in malaria endemic areas, despite improvements in the implementation of effective control measures. Each year, there are an estimated 219 million clinical malaria cases and 435,000 deaths worldwide, the majority of which occur in sub-Saharan Africa [1]. Approximately 60% of the Ethiopian population live in malaria-endemic areas [2, 3].

Of the *Plasmodium* species that cause human malaria, *Plasmodium falciparum* is the main cause of severe malaria and malaria-associated deaths [4]. Severe malaria is differentiated from uncomplicated malaria by a set of diagnostic criteria including clinical and laboratory criteria [5]. The infective load is greater with *P. falciparum* than other malaria species and its propensity to sequester in capillaries leads to increased morbidity [6, 7]. However, the host-parasite factors leading to severe disease or death as compared to uncomplicated disease are not completely understood; these include parasite factors such as genetic diversity and multiplicity of infection (MOI).

Numerous studies have characterized strains of *P. falciparum* isolates using genetic polymorphisms as markers [8–11]. Merozoite surface protein (*msp*) genes are the most commonly used markers of *P. falciparum* due to their high polymorphism. MSP-1 and MSP-2 are *P. falciparum* blood-stage malaria vaccine targets [12] and are also suitable markers for identification of genetically distinct *P. falciparum* parasite sub-populations. MSP-1 is major surface protein encoded by *msp*-*1* on chromosome 9, which contains 17 blocks of sequences flanked by conserved regions. It plays a major role in erythrocyte invasion [13] and is targeted by immune responses [14]. Block 2, which is the most polymorphic part of *msp*-1, is grouped under three allelic families of K1, MAD20 and RO33 [15]. MSP-2 is a glycoprotein encoded by the *msp*-*2* gene located on chromosome 2 and is composed of five blocks of which the central block is the most polymorphic. The *msp*-*2* block 3 alleles are grouped into two allelic families, FC27 and IC3D7 [16]. Genotyping polymorphic regions of *P. falciparum* are important to determine diversity and multiplicity of *P. falciparum* infection [17]. Genetic diversity of *P. falciparum* populations and MOI vary according to the intensity of transmission, outcome of infections and age in different geographical regions [18]. In areas of high malaria transmission, parasite diversity and MOI are increased [4, 18].

Some specific *P. falciparum* genotypes have been associated with severe malaria in epidemiological studies [19]. Severe malaria has also been associated with highly polymorphic parasites [10] and multiclonal parasites [11]. In comparison, other studies have observed a lower frequency of multiclonal infections in *P. falciparum* isolates from patients with severe malaria [20, 21]. Only a few studies have reported on the genetic diversity and clonality of *P. falciparum* in Ethiopia [22–24]. These studies have investigated isolates from uncomplicated symptomatic malaria. Thus, investigation of the *msp*-*1* and *msp*-*2* genes from isolates of patients with severe malaria in Ethiopia is important to obtain knowledge about parasite-factors associated with virulence in Ethiopia. This study aims to explore whether *P. falciparum* genetic diversity and multiclonality are associated with disease severity and age of patients in Gublack area, northwest Ethiopia.

## Methods

### Study site

**Fig. 1:**
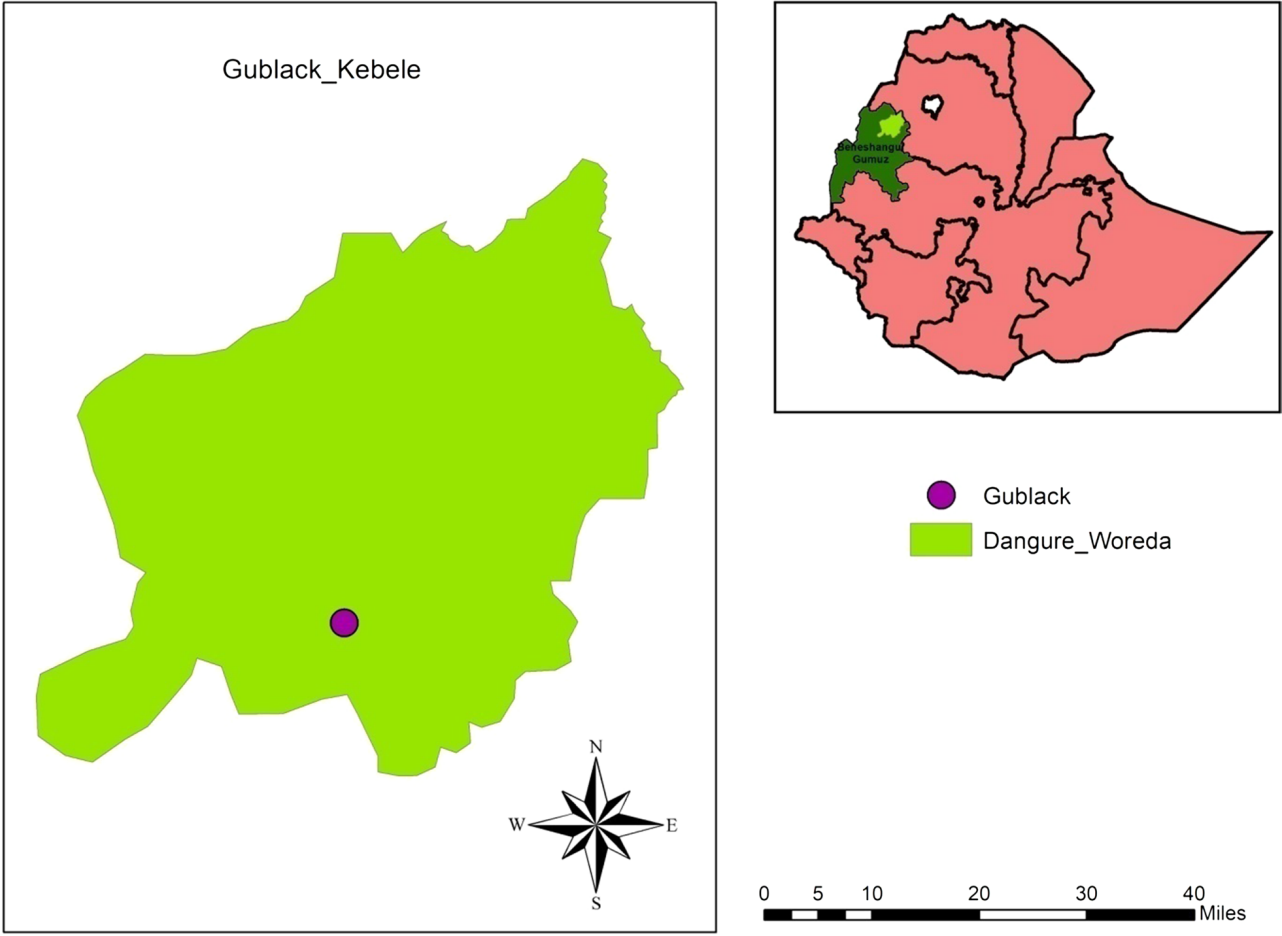
Map of the sample collection area, Gublak, North West Ethiopia. Purple colour indicated the sampling site

A cross sectional health facility-based study was carried out in Gublak Health Center, Dangure district, Benishangul-Gumuz Regional State northwest Ethiopia (Fig. 1). The study area is located 589 kms from Addis Ababa and has a catchment population of estimated 12,088 inhabitants. The area is located at 11° 18′ N latitude, 35° 99′ E longitude at an altitude of 886 m above sea level. *P. falciparum* is the predominant species and *Anopheles arabiensis* is the major vector. The Benishangul-Gumuz region had the highest risks of malaria compared to other regions, and also malaria burden is higher in-migrant labourers coming from nearby highland areas [25, 26]. Malaria transmission in the area is relatively high with all age groups in the population at risk of the disease [27]. The average 3 years of the district malaria positivity rates reported by microscopy to be 64% (unpublished document).

### Study population and blood samples collection

A total of 118 *P. falciparum* infected blood samples were collected from children and adults presenting to the Gublak Health Center and enrolled into the study from July, 2017 to October, 2017. Patients were classified into uncomplicated and severe malaria according to World Health Organization (WHO) criteria [28]. The inclusion criteria for mild (uncomplicated) malaria cases were fever (axillary temperature ≥ 37.5 °C) or history of fever in the previous 24 h, malaria density < 100,000 parasites/µL and absence of danger signs or evidence of severe malaria. Severe malaria cases were defined by additional symptoms such as: convulsion, clinical jaundice, respiratory distress, hyperparasitaemia (> 100,000 asexual parasites/µL) and severe anemia (hemoglobin level < 5 g/dL). Severe malaria patients were admitted to the health center and treated according to the national treatment guidelines [29].

Capillary blood was obtained from a finger prick from each patient and used to prepare thick and thin blood smears. Thick and thin blood smears were air dried and stained with 10% fresh Giemsa for 15 min. All slides were examined by two independent laboratory technicians to determine malaria species and the parasite density. In case of discordance the slides were read by a third laboratory technician. The density of parasitaemia was expressed as the number of asexual parasites per microlitre of blood, assuming a leukocyte count of 8000/µL of blood [30]. Blood smears were considered negative when no parasites were present after the examination of 200 oil immersion fields on a thick blood film. After consent was obtained, the haemoglobin concentration was measured using a portable spectrophotometer (HemoCue^®^, Angelholm, Sweden). The blood was spotted onto Whatman 903^®^ filter paper (Schleicher & Schuell Bio Science, Keene, NH 03431, USA), air-dried and individually placed into a plastic bag with silica gel. These dried blood spots were transported and stored to the Malaria Research Laboratory (Ethiopian Public Health Institute) at Addis Ababa, Ethiopia.

### Extraction of parasite DNA and genotyping of *Plasmodium falciparum msp*-*1* and *msp*-*2* genes

Parasite DNA was extracted from the blood spots collected on filter papers using the Chelex-100^®^ (Bio-Rad Laboratories CA) method [31], with a final volume of 200 µL for each sample and storage at − 20 °C until it was used for the amplification reaction. Allelic genotyping of the *P. falciparum* merozoite surface protein 1 (*msp1*) (block2) and 2 (*msp2*) (block 3) was carried out as previously described [22]. Monoclonal infections were defined by detection of a single PCR fragment for each locus, with polyclonal infection defined if more than one fragment was identified. Multiplicity of infection (MOI) was defined as the average number of different genotypes per infected patient.

### Statistical analysis

Data were entered and analysed using SPSS version 20 (SPSS Inc. Chicago, IL, USA). The *msp*-1 and *msp*-2 allelic frequencies were calculated. The mean multiplicity of infection (MOI) was calculated for *msp*-1, *msp*-2 and overall. The mean MOI was compared between uncomplicated and severe malaria cases using the non-parametric Mann–Whitney U test. The proportion of alleles observed at each locus within each group were compared using Chi-square test statistic. Spearman’s rank correlation coefficient was used to assess the relationships between continuous variables. A p-value less than 0.05 was considered significant.

### Ethical consideration

The study received ethical approval from the Scientific and Ethical Review Office (SERO) of the Ethiopian Public Health Institute (EPHI). Written consent was obtained from the patients and guardians, and malaria positive individuals were treated in accordance with the national malaria guidelines [29].

## Results

### Demographic and parasitological data

The study population comprised 118 microscopically confirmed *P. falciparum* patients of whom 95 (80.5%) had uncomplicated malaria and 23 (19.5%) severe malaria. The major clinical manifestations of the patients are shown in Table 1. The geometric mean parasite density was higher in severe malaria compared to uncomplicated malaria patients (3638 vs. 116,502 parasites/μL), and this difference was statistically significant (p = 0.01). Bed net coverage was 24.6% (29/118) among the confirmed malaria cases.

**Table 1:** Demographics and clinical features of uncomplicated and severe malaria patients in Gublak, North West, Ethiopia

Characteristic	Uncomplicated malaria (n = 95)	Severe malaria (n = 23)
Age, years (mean ± SD)	16.1 ± 9.5	17.0 ± 10.9
Sex ratio (male/female)	0.86 (44/51)	1.6 (14/9)
Axillary temperature, °C (mean ± SD), °C	37.6 ± 0.36	38.2 ± 0.83
Geometric mean parasitaemia, /µL	5724	59,214
Hyperparasitaemia, > 100,000 parasites/µL (n (%))	0 (0.0%)	19 (82.6%)
Convulsions (n (%))	0 (0.0%)	3 (13.0%)
Clinical jaundice (n (%))	0 (0.0%)	3 (13.0%)
Baseline haemoglobin, g/dL (mean ± SD)	11.8 (± 1.8)	8.6 (± 1.7)
Severe anaemia, < 5 g/dL (n (%))	0 (0.0%)	2 (8.7%)

*SD* standard deviation; µL = microlitre

### Allelic frequency, genetic diversity and multiplicity of infection of *P. falciparum* in uncomplicated and severe malaria patients

A total of 180 samples positive for *P. falciparum* were genotyped using MSP1 (block 2) and MSP2 (block 3) by nested PCR and the magnitude of alleles were classified based on their fragments size (Additional file 1: Figure S1; Additional file 2: Figure S2). Of these, 85 (72%) and 95 (80.5%) were positive for MSP1 and MSP2, respectively. Among the msp-1 isolates, the proportion of K1, MAD20 and RO33 allelic families were 54 (45.8%), 26 (22.0%) and 32 (27.1%), respectively. And the *msp*-*2* alleles, the frequency of FC27 allelic family was 58.5% (69/118) and IC/3D7 type was 49.2% (58/118).

The distribution of detected *msp*-*1* and *msp*-*2* allelic families in uncomplicated and severe malaria patients is presented in Table 2. The total number of detected alleles in uncomplicated malaria patients were 19 and 22 for *msp*-*1* and *msp*-*2,* respectively. In severe malaria patients, a total of 11 different alleles were detected for *msp*-*1* and 13 different alleles for *msp*-*2*. Of the 118 *P. falciparum* isolates, 76 (64.4%) harboured more than one parasite genotype (Table 4). Among the multiple infections, 57 (60.0%) and 19 (80.6%) were detected in uncomplicated malaria and severe malaria cases, respectively. Neither *msp1* nor *msp2* allelic families were associated with severe malaria cases (Table 2).

**Table 2:** Allelic frequency of *msp*-1 and *msp*-2 in uncomplicated and severe malaria patients in Gublak, North West, Ethiopia

Allele	Uncomplicated malaria (n = 95)			Severe malaria (n = 23)			p-value*
	n (%)	Fragment size (bp)	No. of alleles	n (%)	Fragment size (bp)	No. of alleles	
MSP-1							
K1	30 (31.6)	110–350	10	2 (8.7)	110–250	4	
MAD20	7 (7.4)	130–350	6	2 (8.7)	150–300	4	
RO33	13 (13.7)	150–200	3	5 (21.7)	150–200	3	
Total	65 (68.4)		19	20 (86.9)		11	0.015
MSP-2							
FC27	31 (32.6)	250–550	9	6 (26.1)	250–500	7	
IC/3D7	19 (20)	300–700	13	7 (30.4)	300–500	6	
Total	73 (76.8)		22	22 (95.7)		13	0.138
Overall MOI			2.0			3.0	

*n* number of positive individuals, *MOI* multiplicity of infection, *total* total number of fragments and distinct clones

* p-value calculated by Chi-square test

The overall mean multiplicity of infection was 2.2 (95% CI 1.99–2.42). The mean MOIs for uncomplicated and severe malaria patients were 2.0 (95% CI 1.79–2.23) and 3.0 (95% CI 2.4–3.59), respectively, which was statistically significant (*p *= 0.001). The mean MOI was highest (2.6, 95% CI 2.06–3.11) among the youngest age groups (< 5 years old) (Table 3) but this was not statistically significant (*p *= 0.104). The youngest age group had the highest mean parasite density (36,345.3 parasites/µL) compared to that of older age groups (21,334.2 parasites/µL) (Table 4). However, the differences in parasitaemia between these age groups were not statistically significant (*p *= 0.09).

**Table 3:** Distribution of *Plasmodium falciparum* of *msp*-1 and *msp*-2 allelic families by age

Allele	Age group (years)			
	0–5, n (%) (n = 24)	6–15, n (%) (n = 14)	> 15, n (%) (n = 80)	Total, n (%) (n = 118)
MSP-1				
K1	8 (33.3)	2 (14.3)	22 (27.5)	32 (37.7)
MAD20	4 (16.7)	2 (14.3)	3 (3.7)	9 (10.6)
RO33	2 (8.3)	3 (21.4)	13 (16.3)	18 (21.2)
K1 + MAD20	3 (12.5)	2 (14.3)	7 (8.8)	12 (14.1)
K1 + RO33	1 (4.2)	1 (7.1)	6 (7.5)	8 (9.4)
MAD20 + RO33	2 (8.3)	0 (0.0)	1 (1.2)	3 (3.5)
K1 + MAD20 + RO33	2 (8.3)	0 (0.0)	1 (1.2)	3 (3.5)
Total	22 (91.7)	10 (71.4)	53 (66.2)	85 (100)
MSP-2				
FC27	9 (37.5)	2 (14.3)	26 (32.5)	37 (38.9)
IC/3D7	3 (12.5)	4 (28.6)	19 (23.7)	26 (27.4)
FC27 + IC/3D7	8 (33.3)	6 (28.6)	18 (22.5)	32 (33.7)
Total	20 (83.3)	12 (85.7)	63 (78.7)	95 (100)

*n* number of individuals

**Table 4:** Distribution of multiple genotypes, mean multiplicity of infection, and parasitaemia by age group of *P. falciparum* malaria patients in Gublak, Ethiopia

Characteristics	Age group (years)			
	0–5 (n = 24)	6–15 (n = 14)	> 15 (n = 80)	Total (n = 118)
Mild malaria, n (%)	17 (70.8)	13 (92.9)	65 (81.2)	95 (80.5)
Severe malaria, n (%)	7 (29.2)	1 (7.1)	15 (18.8)	23 (19.5)
Multiple *P. falciparum* genotypes, n (%)	19 (79.1)	9 (64.3)	48 (60.0)	76 (64.4)
Mean MOI	2.6	2.4	2.1	2.2
Mean parasitaemia (number of *P. falciparum* parasites/μL of blood)	36,345.3	20,875.4	21,334.2	24,332.9

## Discussion

Investigating the association between *P. falciparum* genetic profiles and clinical outcome potentially provides important information for predicting disease-related outcomes in malaria endemic areas. This is the first study that has investigated the association between disease severity, and genetic diversity and multiplicity of infection using the two most polymorphic regions of *msp*-*1* and*msp*-*2* in northwestern Ethiopia. The *msp* genes are usually used for *P. falciparum* population genetics in spite of limitations of the impact of human immune selection [32]. The findings suggest that malaria transmission is high in the study area despite efforts of intensive control measures. The authors also identified a greater propensity for multiclonal infections in patients with severe disease.

Associations between the dominant allelic families and disease severity were examined. In *msp*-1, the K1 allelic family was identified in similar proportions in uncomplicated and severe malaria. This differs to previous studies in Senegal where the K1 allelic family had been associated with severe malaria [33]. This difference may be due to differences in malaria endemicity or the small size of the current study, particularly in the severe malaria cases. The prevalence of two *msp*-*2* families were also similar between uncomplicated and severe disease, which is similar to the previous reports elsewhere [34, 35].

The study identified a higher proportion of *msp*-*2* than *msp*-*1*, which is similar to a study conducted in Sudan [9]. The current study found a high genetic diversity in parasites circulating in the study area with a total of 19 and 22 genotypes for *msp*-*1* and *msp*-*2*, respectively. These findings are similar to those study reported from Senegal [36]. The presence of more genetic diversity in the current study area is likely to be an outcome of the presence of more parasite population, that resulted in mixing of genotypes.

The current study also found a high frequency (64%) of patients with multiclonal infections, which is in line with the previous studies from Ethiopia [22] and Sudan [37]. In contrast, studies from West Africa including the Republic of Congo [38] and Nigeria [39], found a lower frequency of patients with multiclonal infections. This variation likely reflects differences in geographic variability, parasite epidemiology and transmission. The frequency of multiclonal infections has also been reported to increase with age until late childhood before declining [40]. The results were consistent with this finding, with multiclonal infections more prevalent in younger children.

The mean number of circulating genotypes in severe malaria patients was higher than from patients with uncomplicated malaria. Similar results have been observed among patients with severe malaria in Uganda [8]. Studies from Madagascar, Gabon and Sudan [20, 34, 35] showed no differences in uncomplicated or severe malaria cases. However, a study from Nigeria reported a low a multiplicity of *P. falciparum* infection in individuals with severe malaria [41]. These differences may also be related to the immune status of the study population, considerable immigrant labourers were observed in the study area. Moreover, this could also be due to the differences in genotyping methods and interpretation of result, and the heterogeneity of the study populations may suggest the inconsistency variability of results between studies.

Age is a key factor involved in the acquisition of immunity against falciparum malaria and has been found to influence MOI [8]. In turn, the low levels of acquired immunity in young children may be a major factor contributing to their vulnerability to control the infection [42, 43]. Although the mean MOI decrease with age was observed in the current study this was not statistically significant. These results complements the findings from Ethiopia [22], Republic of Congo [38], Senegal [44], and south of Benin [45], but contrast with a study from Tanzania [43]. The lack of a significant trend is potentially limited by power due to the relatively small number of patients younger than 15 years. The high level of malaria transmission in the region may be expected to lead to a higher risk of severe malaria in younger patients where immunity is lower [9], however, the mean age of the patients with uncomplicated and severe malaria was similar.

Bed nets are an important tool for prevention of the malaria vector and are widely used in malaria endemic areas of the Ethiopia. Reduced MOI has observed to be associated with increased ITN use, which is consistent with being an indicator of transmission intensity [46]. The present study found a low utilization of bed nets (24.6%), which might be due to the location of the study area, where there are mechanized farms with migrant labourers sleeping in open fields or temporary shelters [25]. This poor uptake of bed nets might be an important factor contributing to the increased the MOI, with labourers at high risk of developing malaria infection in this area.

Consistent with previous studies, the current study found no association between the frequency of specific *P. falciparum* alleles and disease severity [12, 35]. Limitations in the current study include the small number of patients with severe disease and the use of nested PCR instead of microsatellites or DNA sequencing which could potentially underestimate genetic diversity. Furthermore, with the rapid temporal changes in parasite density and maturity due to parasite sequestration, a single peripheral blood sample may not reveal the full complexity of the parasite population harbored by individuals [47]. In addition, low frequency alleles at the time of blood sampling may not have been detected by nested PCR. Future studies need to be designed to take large sample sizes and use more robust techniques to study the relationship between genetic diversity and malaria severity.

## Conclusion

The present study demonstrates a high multiplicity of *P. falciparum* infections in severe malaria isolates in Gublack, North West Ethiopia. While the MOI was highest among young children, there was no significant difference between MOI amongst age groups. The high MOI and diversity of *P. falciparum* populations in this region could be related to the low utilization of the ITNs. These findings suggested that implementation of customized tools to prevent malaria transmission, by targeting migrant laborers, which substantially improves malaria control in the region. Further longitudinal studies on malaria severity with larger sample sizes in different malaria endemic areas of the country are needed to confirm whether severe infections are more commonly multiclonal across the country.

## Supplementary information

**Additional file 1: Figure S1a.** Gel electrophoresis of K1 MSP-1 allelic families’ lane 1: 100 bp DNA ladder; lane 2: negative; lane 3: 130 bp; lane 4: 300 bp; lane 5, 6, 7: negative; lane 8 and 9: 280 bp; lane 10: negative control. **Figure S1b.** Gel electrophoresis of MAD20 MSP-1 allelic families’ lane 1: 100 bp DNA ladder; lane 2: 150 bp; lane 3: 130 bp; lane 4: negative; lane 5: 180 bp; lane 6: 130 bp; lane 7 and 8: negative; lane 9: 180 bp; lane 10: 150  bp. **Figure S1c.** Gel electrophoresis of RO33 MSP-1 allelic families’ lane 1: 100 bp DNA ladder; lane 2 and 3: 150 bp; lane 4: 180 bp; lane 5: negative control; lane 6 and 7: 150 bp.

**Additional file 2: Figure S2a.** Gel electrophoresis of FC27 of MSP-2 allelic families, lane 1: 100 bp DNA ladder; lane 2: 400 bp; lane 3: 350 bp; lane 4: 350 bp; lane 5: 380 bp; lane 6: 380 bp; lane 7: 320 bp; lane 8: negative; lane 9: 300  bp; lane 10: negative; lane 11: negative control. **Figure S2b.** Gel electrophoresis of IC/3D7 MSP-2 allelic families’ lane 1: 100 bp DNA ladder; lane 2: 400 bp; lane 3: 380–500 bp; lane 4: negative; lane 5: 380–520 bp; lane 6: 380 bp; lane 7: 300 bp; lane 8: 300 bp; lane 9: negative control.

## Data Availability

The datasets analysed for this study are available from corresponding author on reasonable request. All relevant data within the paper.

## Abbreviations

DNA: deoxy-ribonucleic acid
MSP: merozoite surface protein
PCR: polymerase chain reaction
ITN: insecticide-treated bed net
bp: base pair
MOI: multiplicity of infection
