# Supplementary figures and images for: Genetic diversity of Plasmodium falciparum isolates from patients with uncomplicated and severe malaria based on msp-1 and msp-2 genes in Gublak, North West Ethiopia
Source: Malar J. 2019 Dec 10;18:413. doi: 10.1186/s12936-019-3039-9 (PMC6905089; doi:10.1186/s12936-019-3039-9)

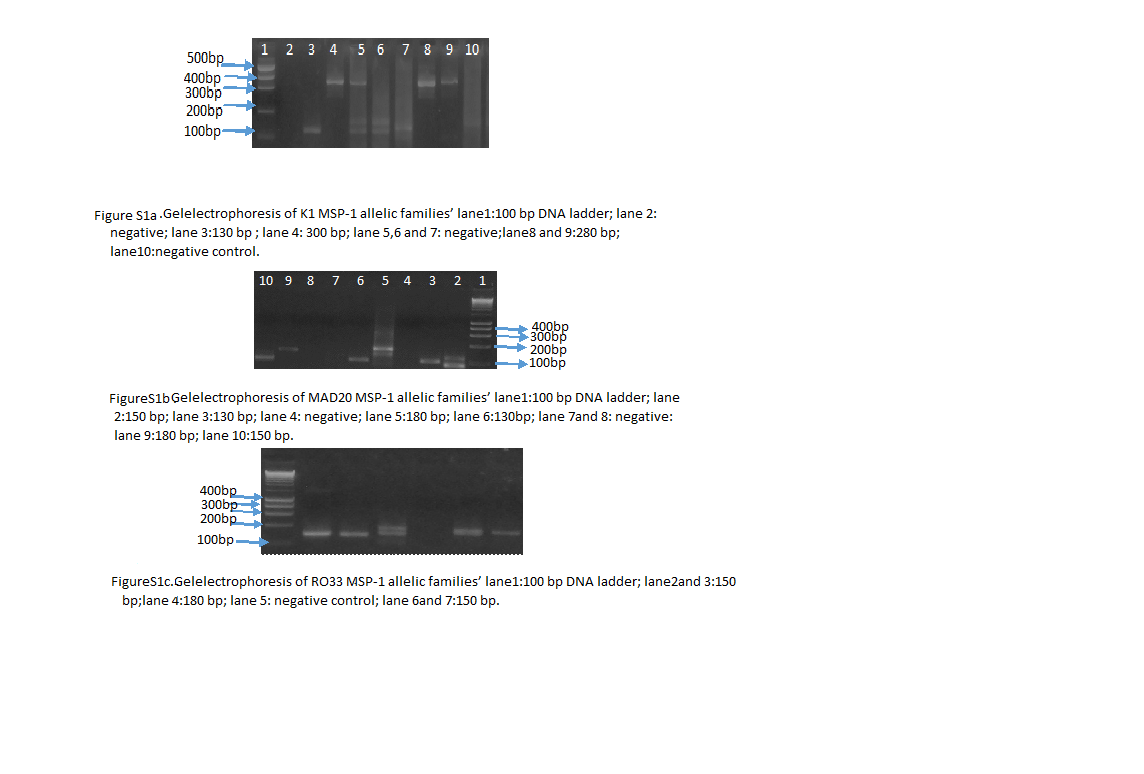

Supplement: Supplementary file 1 — Additional file 1: Figure S1a. Gel electrophoresis of K1 MSP-1 allelic families’ lane 1: 100 bp DNA ladder; lane 2: negative; lane 3: 130 bp; lane 4: 300 bp; lane 5, 6, 7: negative; lane 8 and 9: 280 bp; lane 10: negative control. Figure S1b. Gel electrophoresis of MAD20 MSP-1 allelic families’ lane 1: 100 bp DNA ladder; lane 2: 150 bp; lane 3: 130 bp; lane 4: negative; lane 5: 180 bp; lane 6: 130 bp; lane 7 and 8: negative; lane 9: 180 bp; lane 10: 150 bp. Figure S1c. Gel electrophoresis of RO33 MSP-1 allelic families’ lane 1: 100 bp DNA ladder; lane 2 and 3: 150 bp; lane 4: 180 bp; lane 5: negative control; lane 6 and 7: 150 bp. [file 12936_2019_3039_MOESM1_ESM.bmp]

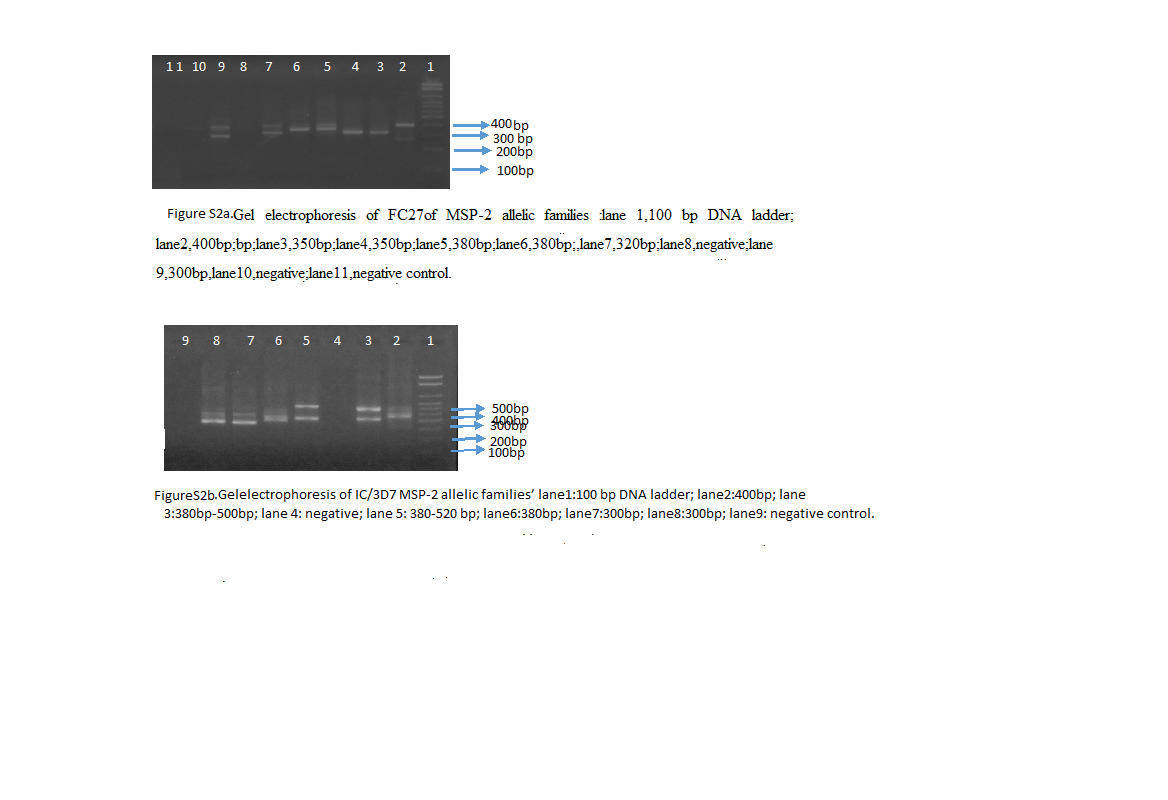

Supplement: Supplementary file 2 — Additional file 2: Figure S2a. Gel electrophoresis of FC27 of MSP-2 allelic families, lane 1: 100 bp DNA ladder; lane 2: 400 bp; lane 3: 350 bp; lane 4: 350 bp; lane 5: 380 bp; lane 6: 380 bp; lane 7: 320 bp; lane 8: negative; lane 9: 300 bp; lane 10: negative; lane 11: negative control. Figure S2b. Gel electrophoresis of IC/3D7 MSP-2 allelic families’ lane 1: 100 bp DNA ladder; lane 2: 400 bp; lane 3: 380–500 bp; lane 4: negative; lane 5: 380–520 bp; lane 6: 380 bp; lane 7: 300 bp; lane 8: 300 bp; lane 9: negative control. [file 12936_2019_3039_MOESM2_ESM.bmp]
